# Correction: Li et al. Community-Level Phenotypic Adaptations of Small Mammals Under Rain-Shadow Dynamics in Baima Snow Mountain, Yunnan. *Animals* 2026, *16*, 91

**DOI:** 10.3390/ani16213407

**Published:** 2026-10-29

**Authors:** Yongyuan Li, Guangzhi Chen, Mengru Xie, Yihao Fang, Feng Qin, Wenyu Song

**Affiliations:** 1 Vector Laboratory, Institute of Pathogens and Vectors, Yunnan Provincial Key Laboratory for Zoonosis Control and Prevention, Dali University, Dali 671000, China; 2 Institute of Eastern-Himalaya Biodiversity Research, Dali University, Dali 671003, China

In the original publication [1], there was a mistake in Figure 4 and Table 3 as published. Upon re-checking, we found an error in the calculation method for the following: 1. The calculation of renal characteristics for each species should originally involve computing the mean values of phenotypic variables (RMT, PMT) for each small mammal individual on an individual basis to determine the species-specific trait value. However, in practice, these morphological indicators were calculated using the average values from all measured individuals within each species. 2. In RMT calculations, one should multiply by 10 less; in PMT calculations, one should multiply by 100 less, which led to underestimated values for RMT and PMT in the published results.

Correction: We have recalculated the results for RMT and PMT as shown in the chart below. The corrected Figure 4 and Table 3 appears below.

Text Correction

There was an error in section “Materials and Methods” 2.4. Environmental Variables and Model Fitting, paragraph 1. Bio2 was mistakenly retained in the text.

Correction: Out of the 19 bioclimate variables, we excluded those indicating temporal temperature variability but retained only those representing differences between hot and cold conditions; hence, Bio2 (average diurnal temperature range) should be deleted.

An item of information requires an update in section “Results” 3.4, Relations Between Water Availability and Renal Characteristics, paragraph 1. As we recalculated the linear regressions of RMT and PMT against AI separately, the results should be updated to report these new values.

Correction: The explanatory effects for AI against both RMT (adj*R*^2^ = 0.357, *p* = 0.024) and PMT (adj*R*^2^ = 0.463, *p* = 0.007) were strong and significant (Figure 4; Table 3).

The authors state that the scientific conclusions are unaffected. These corrections were approved by the Academic Editor. The original publication has also been updated.

## Figures and Tables

**Figure 4:**
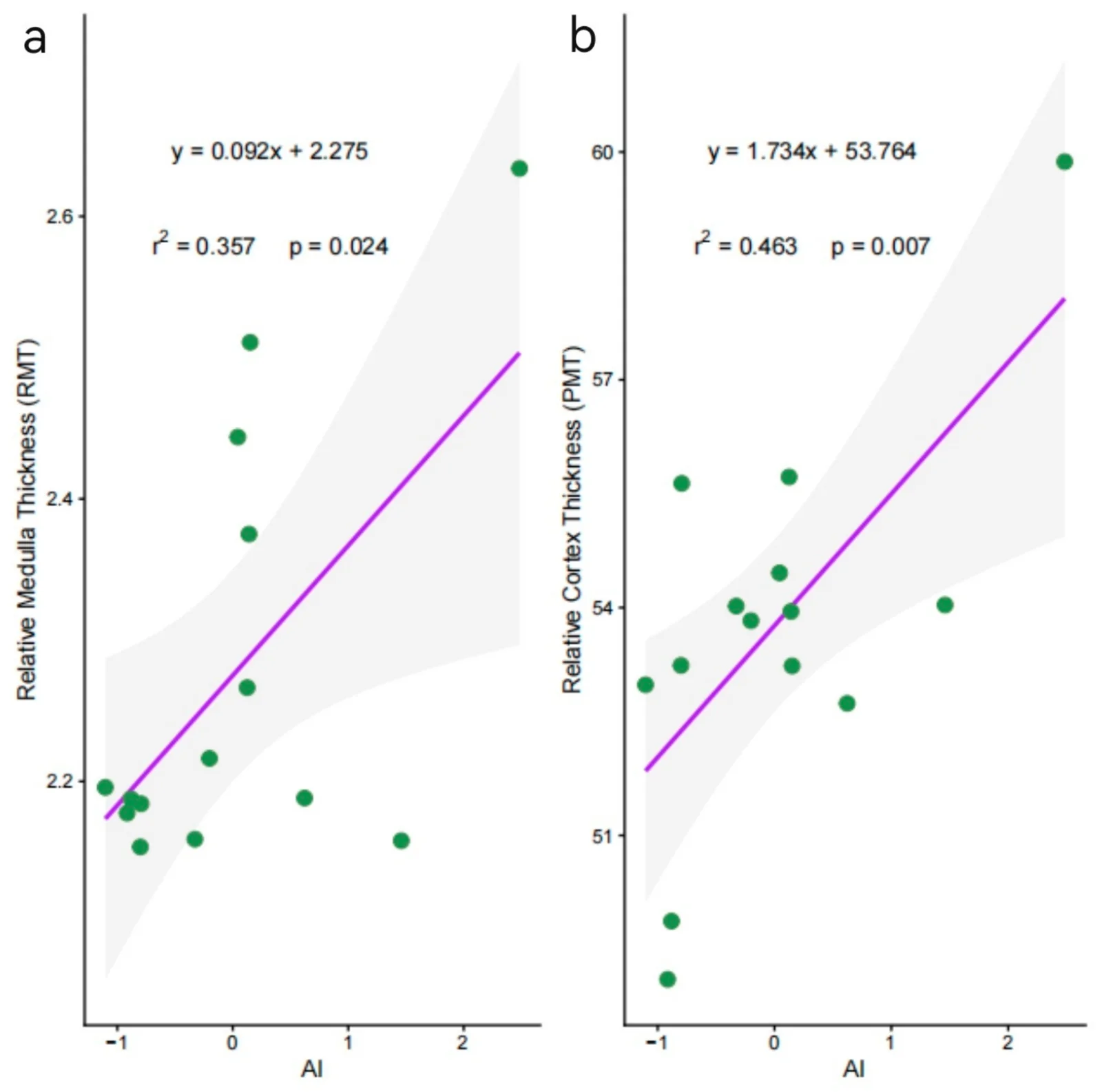
Linear regression relationship between (**a**) relative medullary thickness (RMT) and (**b**) medullary thickness percentage (PMT) as a function of drought index (AI), obtained using ordinary least squares (OLS) method. The higher the AI value, the greater the environmental humidity. The green dots represent the distribution of community weighted mean (CWM); the purple solid line indicates a significant linear relationship, and the shaded areas denote the 95% confidence interval.

**Table 3:** Ordinary least squares (OLS) linear regression results of relative medullary thickness (RMT) and percent medullary thickness (PMT) against aridity index (AI).

Phenotypic Variables	Intercept	Estimate	*SE*	*T*	adj*R*^2^
RMT	2.275 ***	0.092 **	0.0356	2.584	0.357
PMT	53.764 ***	1.734 **	0.539	3.219	0.463

***: *p* < 0.001; **: *p* < 0.01.
